# Evidence for Shaping of Light Chain Repertoire by Structural Selection

**DOI:** 10.3389/fimmu.2018.01307

**Published:** 2018-06-22

**Authors:** Adar Toledano, Yuval Elhanati, Jennifer I. C. Benichou, Aleksandra M. Walczak, Thierry Mora, Yoram Louzoun

**Affiliations:** ^1^Department of Mathematics, Gonda Brain Research Center, Bar Ilan University, Ramat Gan, Israel; ^2^Joseph Henry Laboratories, Princeton University, Princeton, NJ, United States; ^3^Laboratoire de Physique Théorique, UMR8549, CNRS and Ecole Normale Supérieure, Paris, France; ^4^Laboratoire de physique statistique, UMR8550, CNRS, UPMC and Ecole normale supérieure, Paris, France

**Keywords:** deep sequencing, B cell receptor, light chain, selection, rearrangement

## Abstract

The naïve immunoglobulin (IG) repertoire in the blood differs from the direct output of the rearrangement process. These differences stem from selection that affects the germline gene usage and the junctional nucleotides. A major complication obscuring the details of the selection mechanism in the heavy chain is the failure to properly identify the D germline and determine the nucleotide addition and deletion in the junction region. The selection affecting junctional diversity can, however, be studied in the light chain that has no D gene. We use probabilistic and deterministic models to infer and disentangle generation and selection of the light chain, using large samples of light chains sequenced from healthy donors and transgenic mice. We have previously used similar models for the beta chain of T-cell receptors and the heavy chain of IGs. Selection is observed mainly in the CDR3. The CDR3 length and mass distributions are narrower after selection than before, indicating stabilizing selection for mid-range values. Within the CDR3, proline and cysteine undergo negative selection, while glycine undergoes positive selection. The results presented here suggest structural selection maintaining the size of the CDR3 within a limited range, and preventing turns in the CDR3 region.

## Introduction

The diversity of immunoglobulins (IGs) is essential for the function of the adaptive immune system. The IG repertoire is shaped first by the V(D)J recombination processes, and then by selection forces. The rearrangement mechanism determines which genes are combined, as well as the makeup of the junction. Bone marrow and peripheral selection alter this initial repertoire to produce the naïve repertoire observed in the peripheral blood. The repertoire is then further shaped by antigen driven selection to produce the memory repertoire.

The diversity of the IG heavy chain has been studied extensively, like that of the T cell beta chain [see Ref. (1) for review]. It has been shown that much of the diversity originates from the V–D and D–J junctions (2). Current methods to estimate the identity and position of DH are inaccurate for short DH genes (3). Errors in the identification of DH can be erroneously considered as nucleotide addition or deletion. Moreover, in short D genes, the V–D and D–J junctions can overlap and introduce another layer of ambiguity. Here, we focus on the less studied IG light chain to study the roles generation and selection have in establishing functional diversity. An added benefit of studying light chain diversity is that with no D gene inside the CDR3, the junction diversity is more readily separated into contributions from gene selection, and from N insertions (4, 5).

We analyze here the kappa light chain locus (IGK), as the Lambda locus (IGL) has fewer germline genes, and as such has a more limited variability.

Counting all possible V and J choices, deletions, and insertions leads to a vast potential diversity. However, multiple lines of evidence now support that the repertoire is limited:

- The choice of IGKV and IGKJ genes in IGK rearrangement is not completely uniform and preferential use of several genes has been shown (5).
- The IGK polymorphism across individuals has been shown to be much lower than the heavy chain IGH in humans (5, 6).
- Recent results have shown that surprisingly over 20% of light chains CDR3 peptide sequences (out of 700,000 IGL sequences) were “public” (i.e., shared by two or more individuals). Moreover, full length IGL protein sequences (VJ recombinants) are also frequently shared. In a recent study, public rearrangements made up of 60.2% of the total sequenced IGK rearrangement (7).

These results together suggest that non-uniform rearrangement, biased junction formation, structural selection, and functional selection can shape the repertoire (5, 8–12). However, the relative contribution of these different mechanisms in the light chain repertoire has never been studied. We here study the extent and origin of IGK diversity using sequences of the recombined gene obtained from blood samples for humans and mice. This observed recombined repertoire is shaped by the rearrangement mechanism and by selection (either positive or negative). To understand how the repertoire is selected, these two processes must be separated.

We do not delve here on the V and J usage and their correlation. Those have been argued based on both theoretical and experimental results to be induced by the receptor editing mechanism (13–16).

The generation and the selection processes are stochastic in nature, with different recombined peptides having different likelihoods of being generated and selected. We use statistical models, where the probability of assigning each observed sequence to appropriate germline genes and junction sequences are computed, to infer and disentangle the two processes. We find that structural selection strongly shapes the observed light chain repertoire.

We have used similar models on T cells and heavy chain B cells (17, 18). Here, these models enable us to study the variability of the IGK light chain during the generation and initial selection stage of B cells. The IGK samples, sequenced from healthy donors and from transgenic mice, are first divided into functional and non-functional recombined genes. The functional sequences are in-frame (IF) and with no stop codon, and as such code for a peptide that can potentially be the light chain of the IG. Out-of-frame (OF) sequences, on the other hand, underwent recombination that resulted in some of the conserved codons of the J template to be out of their normal reading frames and thus lack essential conserved amino acids when translated. They sometimes also have stop codons, which prevent them from being fully translated. These OF sequences, having never coded for any protein, did not undergo selection and represent the results of the raw generation process. By comparing the statistics of the OF sequences (the generation process statistics), to the IF sequences, selection can be inferred (see Materials and Methods). We have studied Rapid Amplification of cDNA Ends (RACE)-based cDNA sequences of human and mouse light chains. The human light chains were taken from peripheral blood and were separated into naïve and memory cells. The mouse cells were separated into blood and bone marrow cells (see Table S1 in Supplementary Material for details).

## Materials and Methods

### Generation Model

The V(D)J recombination process involves a random number of insertions and deletions, and often produces OF sequences. These sequences code for non-functional proteins and can still appear in a blood sample, if the second chromosome in the cell underwent a successful recombination. In such cases, the sequences experienced no selection and owe their survival to the receptor expressed by the other chromosome. Thus, they provide us a glimpse into the pure generation process. We used these OF sequences to infer the statistics of the V(D)J recombination process.

Each observed sequence can be the result of a number of scenarios that include different initial gene choices, followed by a variable number of deleted and inserted base pairs. Estimating the probability of a sequence can be done by summing over all the different possible scenarios for producing a given sequence, weighting each scenario by its probability. Each scenario’s probability (Pgen) is calculated using a probabilistic generation model of the form P(V,J)P(delV\V)P(delJ\J)P(ins). In brief, the various factors account for the probabilities of uncorrelated events leading to a specific VJ rearrangement: choice of which gene segments to recombine P(V,J), probability of the number of deletions from the ends of the V and J genes at the junctions P(delV|V) and P(delJ|J), choice of number of nucleotides to insert P(ins), as well as factors to account for unequal nucleotide preference in the inserted sequences. This type of model was used before to infer the generation process of heavy chain in B cells and beta chain in T cells.

Here, we used the Baum–Welch algorithm to efficiently infer the parameters of the generation model (18). In short, by reformatting the generation model as a Markov model, we used the forward–backward algorithm once per sequence, then summing over all sequences to update the model parameters. This is a dynamic programming approach that bypasses the need to enumerate all possible recombination scenarios.

### Selection Model—Probabilistic Model

The naïve productive sequences (IF and with no stop codon), unlike the non-productive ones, have passed an initial selection process before being admitted to the periphery. We used the productive sequences to learn the selective forces acting on amino acids by comparing how their statistics differ from the raw product of V(D)J recombination learned from the OF sequences.

Using the generation model as a starting point, we infer selection factors *Q* acting on each sequence in the naive repertoire, where *Q* is defined as the fold increase of the probability to see a sequence in the functional repertoire (naive, productive) compared with the previously learned generation probability: *Q* = *P*_post_/*P*_pre_. To infer those factors, we use a factorized model *Q* = *q*(V,J)*q*_L_Π*q_i_*_;_*_L_*(*a_i_*), where we assume that selection acts independently on the V,J gene choice [through factor *q*(V,J)], the length *L* of the CDR3 sequence (through factor *q_L_*), and on each of the amino acids *a_i_* at positions 1 ≤ *i* ≤ *L* between the conserved cysteine near the end of the V gene and the conserved tryptophan within the J gene [through factors *Q_i;L_*(*a_i_*)]. We use an expectation–maximization procedure to update the selection factors until convergence (1).

### Study Subjects

For the human data, 12 apparently healthy adult subjects (3) were recruited for high-throughput sequencing using the 454 platform. Two 45-ml blood draws were collected in heparin tubes from each subject at a single time point. Mononuclear cells were isolated using Ficoll-Paque Plus (GE Healthcare), and then sorted by flow cytometry into naïve (CD20+, CD27−) and memory (CD20+, CD27+) populations. Informed consent was obtained from all donors. This work was performed in accordance with an IRB-approved protocol at Pfizer, Inc.

For the mouse data, blood and bone marrow RNA was extracted from healthy C57BL/6J mice using Qiagen RNAeasy Mini (19). RNA was provided as input to Clonetech SMARTer 5′RACE reactions, using murine IgK specific primers. Amplicons received Roche 454 adaptors with DNA barcode multiplex identifiers, and then sequenced with Titanium chemistry. The human and mouse data used here are based on previous publications (3, 20).

### Target Amplification and 454 Sequencing

Unbiased amplification of repertoires was performed by 25 cycles of 5′RACE, using individual isotype-specific reverse primers. Primers were optimized for efficiency, fidelity, and completeness of repertoire recovery by informatics screening, gel-analysis, and high-throughput sequencing of recovered products. The degree of germline-dependent amplification bias was assessed by comparing amplified products of stimulated naïve B cell pools to direct sequencing of the same pools. Cycle-dependent effects on diversity estimates were evaluated by high-throughput sequencing. All products received multiplex identifiers (barcodes) to allow unambiguous identification of all products by sequence analysis in subsequent processing steps. Multiplex identifiers differed by at least three base pairs from any other multiplex identifier sequence, and only reads with exact-matches were included in the analysis. Products were sequenced with 454 Titanium. Sequencing quality was assessed by keypass control. Sample QC was confirmed by demultiplexing and VK segment genotype. Sequencing depth was determined by diversity estimate rarefaction and simulations of germline-profile stabilization as a function of sequencing depth. A detailed validation of the sequencing methodology has been provided previously (12).

### V–J and Clone Detection Pipeline—Deterministic Model

We detected clones by clustering together sequences with similar CDR3 sequences (further explained below), to minimize the effect of potential biases in sequence copy numbers.

Specifically, sequences were grouped into clones using a two-step approach. First, we assigned each sequence V and J germline genes by running the IgBLAST tool (21) against human and mouse germline sequence databases (appropriately). Next, to count the clones, we grouped all sequences according to their V and J usage as well as the distance between V and J, since SHMs usually do not produce insertions or deletions of nucleotides (22). Thus, every clone emerging from the same founder cell should have the same distance between V and J. We then took all of the sequences with the same V–J and the same distance between V and J and grouped them using a phylogenic approach. All the sequences with an identical V–J and an identical distance were aligned together, using an artificial sequence composed of the germline sequences and gaps between them. The beginning and the end of all sequences of each dataset were trimmed so that all the sequences have same length V and J segments. The sequences of each group are thus already aligned and a phylogenetic tree was built using maximum parsimony (23) and/or neighbor joining (24) methods (from the PHYLIP 3.69 program package). We then parsed this tree with a cutoff distance of four mutations into clones. Thus, a clone was defined as a set of sequences similar to one another, up to a distance of four mutations. These methods were extensively validated in previous studies (1–3, 25–27).

### Sequence Analysis

#### CDR3 Length

We calculate CDR3 length according to the number of amino acids between the conserved cysteine and phenylalanine. We then used the same sequence to compute the total CDR3 molecular mass (MW) using the “peptides” R package (values are rounded up to two digits). We then computed the distribution of CDR3 lengths in AA and in MW, and compared the SD of these distributions in different sets. For the MW relative difference, we calculate the SD of the MW in the IF sequence divided by the SD of the MW in the OF sequence minus 1 (to have 0 represent a state of no selection). The AA length SD ratio was calculated similarly. We did the same thing for the relative difference average of the MW and length.

#### Selection vs. Generation Probabilities

In the *p* − *q* plot, we present the log of the selection factor *q* vs. the log of the generation probability *p*. We computed the Spearman correlation between these two values for the generation probability of VJ pairs and for the probability of a given amino acid in each position and CDR3 length. Formally, we calculated the correlation between the generation probability and selection factors across amino acids where *P_i;L_*(*a_i_*) is the generation probability for amino acid (*a_i_*) in position *i* for length *L* (for maximum length 19, this can be coded with 20*19*19 parameters, some of which are zeros). The *Q_i;L_*(*a_i_*) is the selection factors of the same amino acid, length, and position.

#### Average Selection Factor

To present the selection factors of the different amino acids in the different positions, we averaged all the *q*-values over CDR3 lengths for each codon. Then, we present the results of the log values on a heat map. We also computed the log of the average of the selection affecting all codons translated to the same amino acid as presented in Figures 2 and 3.

## Results

### CDR3 Are Selected to Have a Narrower Distribution

Naive B cells have undergone light chain-dependent selection (28). To study this selection, we first investigated the difference in the light chain CDR3 length distribution before and after selection in naïve and memory repertoires (the naïve pool in the peripheral blood, and the memory pool resulting from germinal center driven selection). The length of CDR3, defined as the number of nucleotides between the cysteine and phenylalanine surrounding the CDR3 [see Ref. (29) for CDRs positions definitions], was analyzed in samples from peripheral human blood that contains naïve and memory cells and mice B cell samples in the blood and bone marrow (see Table S1 in Supplementary Material and Materials and Methods for details).

We used deterministic and probabilistic generation model to compare the OF and IF repertoires. The probabilistic generation model was developed to best fit the OF human light chain samples, and the model was then applied to evaluate the generation probability of the IF naïve light chain repertoire. The validity of this method has been extensively tested (17, 18). For the other human and mice samples (mouse blood, mouse bone marrow, and human memory B cells), where the data were more limited, we only used the deterministic model, where each sequence is assigned the most probable V and J genes and the most probable alignment as estimated by our clone detection pipeline, which was also validated in multiple studies (1, 3, 25). The general features, such as V and J genes, are similar in the deterministic and probabilistic models. Note that we here study generic properties of the B cell receptor repertoire, and our results do not require an extreme sequencing depth or a very low-sequencing error level. Thus, the 454 sequencing used here is precise enough for the current analysis.

For each observed clone, only one sequence (the ancestor of the clone) was analyzed. Multiples conditions were compared. We used the OF sequences as representative the output of the rearrangement process, and compared those to naive cells to study the selection taking place in the bone marrow, or in the periphery prior to antigen exposure. We also used memory cells to test the effect of antigen exposure on the L chain repertoire. Finally, we analyzed mouse bone marrow and peripheral B cells and compared them with mouse OF cells to test again selection within the bone marrow and in the transition to the naive repertoire in the periphery. The probabilistic model was applied to the human naive cells and it thus represents again the selection affecting the naive repertoire, probably prior to antigen exposure.

**Figure 1:**
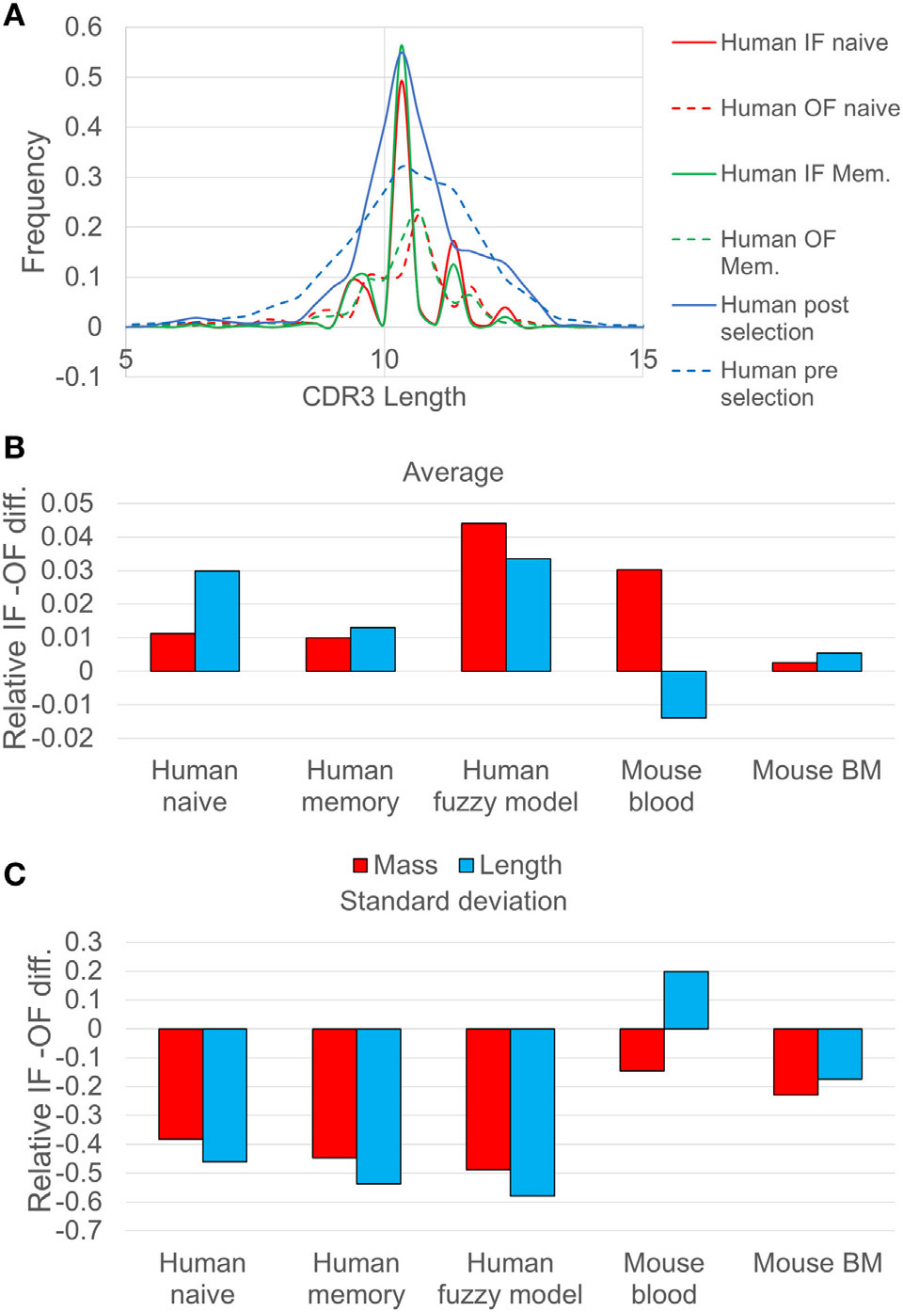
**(A)** Comparison of CDR3 length distribution in human in-frame (IF) and out-of-frame (OF) sequences. The continuous lines represent the IF reads, while the dashed lines represent the OF reads. Pre-selection (IF) and post selection (OF) curves (blue) correspond to human naive sequences analyzed using the probabilistic model, whereas the red curves correspond to the same human naive sequences analyzed using the deterministic model. The CDR3 length distribution is narrower after selection, indicating selection against too long or too short sequences. **(B)** The relative difference between the SD of CDR3 length/mass between IF sequences and OF sequences (the ratio minus 1), for different samples of human and mice. The blue bars represent the CDR3 length ratio, and the red bars represent the calculation of CDR3 mass ratio (the *p* values of the *F*-test are less than 0.001 except from the mouse blood sample which are less than 0.01). **(C)** The same for the average over length/mass of the CDR3, for different samples of human and mice (the *p* values of the *T*-test are less than 0.001 except from the mouse BM and the mouse blood which are less than 0.1).

A comparison between the OF-based stochastic model and the length distribution in the IF naïve human sequences indicates that there is a very weak change in the average length of the CDR3 (Figure 1C). The slightly longer CDR3s in functional sequences are in contrast with previously reported shortening of the heavy chain during development (30). This increase is accompanied by a parallel increase in the total molecular mass.

A more drastic change between the IF and the OF rearrangements is the reduction in the length variance (Figure 1B), indicating selection against short or long CDR3 sequences. A similar result can be observed when comparing the results of the deterministic model (full vs. dashed lines in Figure 1A and appropriate bars in Figures 1B,C). The reduction in the length distribution width is highly significant. The length distribution for mice shows the same trends (*F* test *p* < 1.e−3 for all tests, except for mouse blood where the IF CDR3 lengths are slightly more diverse than OF).

The difference in the human repertoire CDR3 length variance is much larger than in the mouse repertoire. The main reduction in the CDR3 length variability occurs in the human repertoires between the OF and naïve, and not between the naïve and memory, suggesting a pathogen-independent selection for intermediate CDR3 length. While in the mouse repertoire the SD of the length measured in nucleotides did not decrease significantly in the blood, the SD of the total molecular mass of the CDR3 did decrease significantly (*F* test, *p* < 0.01). The difference suggests that in humans, the total mass of the CDR3 is maintained by limiting the CDR3 length variability, in mouse the result is obtained by balancing large and small amino acids within the CDR3. The simplest explanation for the reduction in the light chain mass variability would be structural selection of the shape of the light chain, where too large or small total mass would prevent the binding to the heavy chain or to potential antigens.

### Selection Is Not Sensitive to Codon Identity

**Figure 2:**
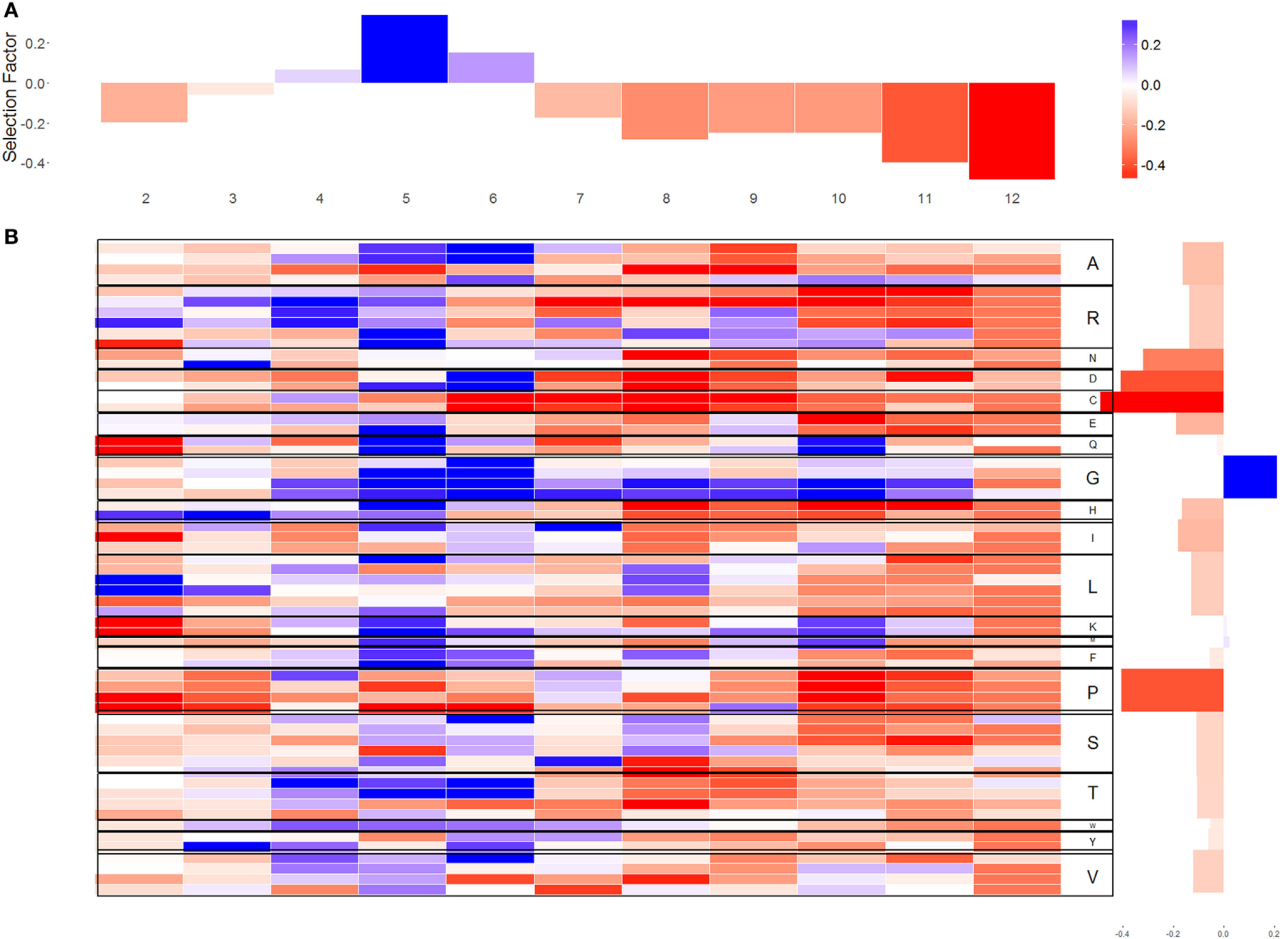
Selection factors for sequences. **(A)** The average of the *q*-values for each location in CDR3 in log scale [positive selection represented by positive values log(*q*) > 0 and negative selection represented by negative values log(*q*) < 0]. **(B)** The *x*-axis represents the different location in CDR3; the *y*-axis represents the different codons. In the right side of the figure, each codon is translated to its amino acid. The blue cells represent log(*q*) values larger than 0 for codon in a certain position. The red cells represent log(*q*) values lower than 0. The white cells indicate that there was no selection.

**Figure 3:**
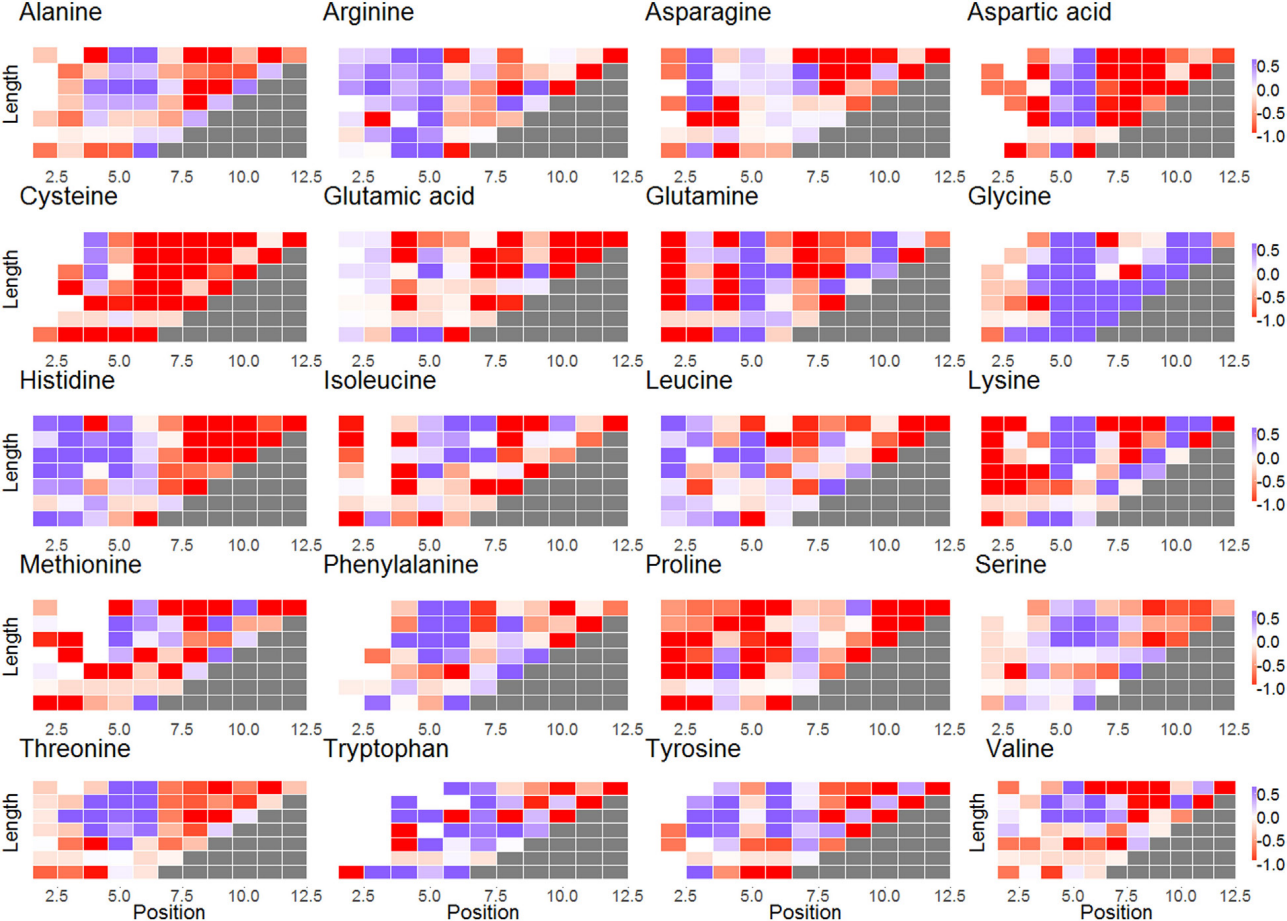
Subplots of selection factors of different lengths for each amino acid. The *x*-axis represents the different locations in CDR3; the *y*-axis represents the different lengths of the CDR3. The blue cells represent log(*q*) values larger than 0 for codon in a certain position. The red cells represent log(*q*) values lower than 0. The white cells indicate that there was no selection.

Beyond the length and size of the CDR3 region, the specific composition of the CDR3 affects its selection and production scores. We have used the human kappa chain probabilistic generation and selection models to estimate selection pressures for amino acids and individual codons (Figures 2 and 3). This is done using selection factors that measure the selection pressures on the different codons or amino acids, for every position and CDR3 length. These are learned from IF data, such that their combined effect amounts to the difference in amino acid usage from the OF sequences (see Materials and Methods for details). For presentation, the factors were averaged over CDR3 lengths (Figures 2A,B), and over codons for the same amino acid (Figure 3). We present the log of the selection factor. Selection factors higher than 1 (log higher than 0—blue values in Figures 2 and 3) represent positive selection (i.e., sequences containing this codon/AA at this specific position are over-represented compared with the expected from the OF sequences), while factors lower than 1 (log lower than 0—red values in Figures 2 and 3) correspond to negative selection.

Different codons coding for the same amino acid have highly similar selection patterns (Figure 2B), suggesting that the selection affecting naïve B cell acts on amino acids, and not on codons. Such selection would agree with structural selection on the formed light chain, instead of a genetic mechanism favoring some specific nucleotides in the junctions (the variance of the log selection factors between the codons coding for the same amino acid is 0.154 and the variance between amino acid is 0.372).

### Selection Favoring Glycine and Against Proline, Cysteine, and Aspartic Acid

Selection patterns differ between amino acids. Cysteine (Wilcoxon test, *V* = 203, *p*-value = 4.618e−15), proline (*V* = 645, *p*-value = 1.746e−13), and aspartic acid (*V* = 773, *p*-value = 2.955e−08) clearly undergo negative selection, whereas glycine (*V* = 4206, *p*-value = 1.168e−06) is under positive selection (in almost all locations along the CDR3) (Figures 2 and 3). In addition, some amino acids such as histidine and arginine have a positive selection in the beginning of the CDR3 and negative selection on the other side. Proline is unique as an amino acid, since its residue (R) is attached to NH atoms. This special structure breaks (spatially) long-peptide chains. Therefore, it is sometimes used in points of sharp folding of proteins (31). Proline may thus undergo negative selection to avoid the curvature and folding. Similar results were observed in the heavy chain (3).

A similar argument may explain selection against cysteine to prevent disulfide bonds, as is also observed in heavy chain (17). The selection in favor of glycine is the precise opposite with a selection for a tiny (the smallest) AA that has very limited interactions with other AA and a limited effect on the shape of the light chain CDR3 region. We currently have no clear model for the negative selection that observed in the aspartic acid, since its properties are highly Ph sensitive, and we cannot determine in which conditions selection shapes the repertoire.

### Selection Is Mainly Positive in Positions 5–6 of CDR3 and Mainly Negative in the Following Positions

Selection is not uniform along the CDR3. The log of the selection factors are close to 0 in the third amino acid of the CDR3 that is outside the binding site of the antigen (−0.032 ± 0.4868). For most amino acids, positions 5 and 6 undergo a significant positive selection, showing a clear deviation in favor of rare amino acids (correlation between the log of the selection factor of position 5 with the AA frequency is 0.329, correlation with position 6 is 0.249), exactly at the beginning of the antigen binding site [5th position—(29)]. From positions 7 to 12, on the opposite site of the binding site, a significant negative selection can be observed for most amino acids apart from glycine and in specific positions also alanine, lysine, and glutamine, suggesting that long sequences are quite restrictive in this area, which ties in with the fact that long CDR3 are generally selected against as discussed above (these positions only exist in long CDR3 that are selected against).

For some amino acids, selection is length and position dependent, while for others, it is almost constant. Specifically, certain amino acids undergo different selection when close to the ends of the CDRs, in contrast to the middle (see, for example, alanine or aspartic acid in Figure 3). Other amino acids have positive or negative selection in almost all lengths and positions (glycine and cysteine and proline, respectively) in agreement with previous results (17). Note that this selection occurs in the naive repertoire, and it is thus probably not driven by pathogens.

## Discussion

Immunoglobulin genes are created in a stochastic V(D)J recombination process that is function independent. The distribution of possible receptors is not uniform; there is large variability in the generation probability of different elements of the rearrangement [e.g., V(D)J choice, junctional composition]. Beyond these differences, there are differences between the naïve repertoire and the one directly emerging from the rearrangement process.

A possible reason for this last difference may be the relation between the biochemical properties of the receptor and its potential binding to antigens. Such binding is mainly associated with the properties of the variable peptide chain of the CDR3. Many of the sequences generated might not code for receptor proteins that could potentially bind antigen. Some form of selection could then act to purify the generated repertoire into the functional one, observed in the naïve pool in the periphery. For example, there could be positive selection for binding self-antigens.

Here, we explored this notion of initial selection by analyzing the difference between the properties of IF and OF light chain rearrangements in naïve and memory repertoires, in human and mouse cells using probabilistic and deterministic generation models. An important advantage of the light chain repertoire analysis is the absence of the D gene, drastically simplifying the rearrangement process.

We have shown that selection acts mainly on the CDR3 rather than on the templated part of the V and J genes. Within the CDR3, selection tends to limit the variance of the CDR3 size in both human and murine repertoires in the transition from the direct rearrangement process to the naïve repertoire. These variances decrease by more than 45% during this transition. Interestingly, while in human light chains, the variance reduction is mainly through the removal of light chains with a low or high number of nucleotides in the CDR3, in mice the reduction is through a change in the distribution of amino acids in the CDR3, making it more restrictive. The reduction in CDR3 length variance was mainly observed between the repertoire emerging from the rearrangement and the naïve repertoire and not between the latter and memory, suggesting the vast majority of the structural selection occurs in the bone marrow, and is not pathogen driven.

In humans, amino acids affecting the structure of the CDR3 region, such as proline, are selected against, while tiny amino acids such as glycine are favored. Similar preferences have been observed in the heavy chain (18).

A correlation has been observed between the production probability of each amino acid and its selection in the transition from rearrangement to the naïve pool, suggesting a long-term evolutionary process favoring some junctional amino acids, which are later further selected within a host. Such a behavior has been previously reported in the heavy chain and T cell beta repertoires (17, 18). Selection does not seem to be affected by the codon used, but it is both position and CDR3 length dependent, for some amino acids. Among most amino acids, 5′ regions have higher selection scores than 3′ regions.

All of these elements strongly suggest structural selection where the proper structure of the light chain, and possibly its binding to the heavy chain are selected for. The main selection step has been reported between the OF and the IF naïve repertoire.

The V and J composition of the light chain are not independent. However, this dependence could be the direct result of light chain editing (replacement of non-functional rearrangement by new rearrangements) (14–16). Moreover, differences in the VJ pairing of IF and OF are expected even in the absence of selection, since IF rearrangement are typically made after OF rearrangement, due to repeated light chain rearrangement in the same chromosome, and as such favor more distal VJ combinations (13).

The difference between IF and OF B cell receptor repertoire was argued to highlight properties of B cell receptors associated with diseases or pathogenic challenges. However, current and other recent results (2, 3, 17, 18, 25, 26, 30, 32–37) highlight that the observed naïve repertoire is very different from the direct result of the rearrangement process. Thus, three different repertoires should be defined:

(1) A repertoire produced from rearrangement during the early pro- and pre-B cells stages in the bone marrow.
(2) A naïve repertoire, which is the result of bone marrow selection mechanisms that may be either antigen dependent or independent, and
(3) A memory and plasma-blast repertoire shaped by antigen and possibly pathogen driven selection.

The difference between the last two repertoires seems to be more limited than the difference between the first two. The next challenge will be to develop models to detect within the structurally selected naïve repertoire, BCRs with a potential functional CDR3. Using statistical models of the naïve repertoire that went through the initial structural selection step, we will be able to detect minute differences that indicate selection by exposure to pathogens.

## Author Contributions

AM performed analysis, wrote part of manuscript, and produced figures. YE designed and performed probabilistic analysis, and wrote part of manuscript. YL designed analysis and wrote part of manuscript. AW and TM helped writing the analysis and designing the probabilistic model. JB performed the BCR bioinformatics.

## Conflict of Interest Statement

The authors declare that the research was conducted in the absence of any commercial or financial relationships that could be construed as a potential conflict of interest.

## Supplementary Material

Table S1 The samples that were used for each model and their quantity. In subsets of both the mouse data and of the human data, we perform the analysis only on the deterministic model because of small sample sizes.
