# Supplementary figures and images for: Evidence for Shaping of Light Chain Repertoire by Structural Selection
Source: Front Immunol. 2018 Jun 22;9:1307. doi: 10.3389/fimmu.2018.01307 (PMC6023962; doi:10.3389/fimmu.2018.01307)

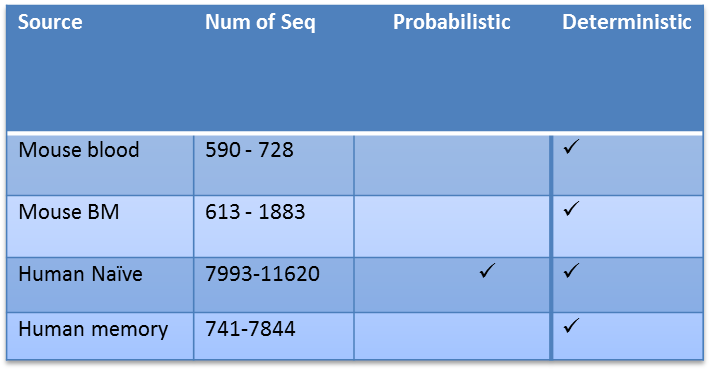

Supplement: Table S1 — The samples that were used for each model and their quantity. In subsets of both the mouse data and of the human data, we perform the analysis only on the deterministic model because of small sample sizes. [file table_1.docx]
